# The burden of antimicrobial resistance in biofilm forming *Staphylococcus* spp. from Vernal Keratoconjunctivitis patients eyes

**DOI:** 10.1016/j.bioflm.2025.100278

**Published:** 2025-04-05

**Authors:** Nelaveni Rupa, Pragnya Rao Donthineni, Sayan Basu, Kotakonda Arunasri

**Affiliations:** a Prof. Brien Holden Eye Research Centre, L V Prasad Eye Institute, Banjara Hills, Hyderabad, 500034, Telangana, India; b Shantilal Shanghvi Cornea Institute, L V Prasad Eye Institute, Hyderabad, 500034, Telangana, India

**Keywords:** *Staphylococcus* biofilms, Antimicrobial resistance, Vernal keratoconjunctivitis, Conjunctival bacteria

## Abstract

Vernal keratoconjunctivitis (VKC) is a chronic allergic ocular surface disease with seasonal recurrences and severe forms showing vision threatening complications. The purpose of the study is to understand the prevalence and diversity of biofilm-forming bacteria and antimicrobial resistance in VKC compared to healthy individuals (HC). For this, conjunctival swab samples were collected from VKC (n = 26) and HC (n = 23), of which culture positive samples were 77 % and 78.26 % respectively. The 16S rRNA gene sequencing revealed a significant increase in bacterial diversity in VKC compared to HC (p < 0.05), identifying 16 and 9 bacterial species, respectively. *Staphylococcus epidermidis* emerged as the predominant bacterium in both groups, with relative abundances of 52.8 % in HC and 30.2 % in VKC (p < 0.001). Biofilm formation was observed in 64.15 % of bacterial species in VKC and 31 % in HC (p < 0.001). Scanning electron microscopy analysis confirmed temporal biofilm formation by *Staphylococcus* spp. in both groups. Minimum inhibitory concentration testing showed that biofilm forming *Staphylococcus* spp. from VKC exhibited multidrug resistance (>2 antibiotics) more frequently than those from HC. Additionally, *Staphylococcus* spp. in VKC demonstrated higher resistance to fluoroquinolones compared to HC. These findings indicate a significantly greater prevalence of biofilm-forming and antimicrobial resistant *Staphylococcus* bacteria in VKC Patients compared with HC.

## Highlights

• Bacterial diversity and biofilm forming bacteria were significantly higher in VKC compared to HC.
• The predominant bacteria observed in the VKC variants were, *S.haemolyticus* in limbal and *S.epidermidis* in palpebral and mixed variants.
• The MIC of tested antibiotics were higher in VKC isolates compared to HC. A greater proportion of VKC *Staphylococcus* isolates formed biofilms and exhibited resistance to more than 2 antibiotics.
• *Staphylococcus* isolates from HC were 100 % sensitive to fluoroquinolones and vancomycin, while some of the VKC isolates exhibited resistance.

## 1 Introduction

Vernal Keratoconjunctivitis (VKC) is a chronic, allergic ocular surface disease that predominantly affects children and young adults, displaying a range of symptoms that include severe itching, redness, and visual disturbances [[1], [2], [3]]. The pathogenesis of VKC is multifactorial, involving environmental allergens and immune response [[4], [5], [6]]. Recent research has indicated that the ocular surface microbiota plays a significant role in immune response and disease progression in ocular diseases. This highlights a complex interplay between the host, environmental factors, and the microbial communities [7,8]

The role of ocular surface bacteria in maintaining the homeostasis in healthy eyes and its association with ocular diseases was studied previously [[9], [10], [11], [12], [13]]. The sporadic reports on the bacterial composition in VKC eyes indicates growth of bacteria such as *Staphylococcus, Escherichia, Micrococcus, Streptococcus, Enterococcus, Pseudomonas, Klebsiella* and *Serratia* from the ocular samples[[14], [15], [16]] These studies have also indicated a culture positivity up to 77 % of the samples used for culturing. In VKC it was shown that specific ocular surface bacteria tend to overgrow, which may contribute to worsening of VKC and may predispose them to develop secondary infections [3]. Notably, *Staphylococcus* species, particularly *Staphylococcus aureus* and *Staphylococcus epidermidis*, have been frequently isolated from the conjunctival sac of VKC patients [17,18]. *Staphylococcus aureus* is known for its pathogenic potential, as it can produce various virulence factors that exacerbate inflammation and contribute to the disease's severity. It was also indicated that the Staphylococcal enterotoxin A and B-specific IgE antibodies trigger and exacerbate allergic conjunctivitis [[19], [20], [21], [22]]. While *Staphylococcus epidermidis* is a part of the normal ocular surface flora, it has the potential to become opportunistic in the setting of dysbiosis or immunological challenges [23]. Additionally, *Staphylococcus* spp. are also known to exhibit virulence by forming biofilms thereby exhibiting resistant to various antibiotics [24]. Other bacteria, such as *Haemophilus influenzae, Pseudomonas aeruginosa* and *Moraxella* species have also been implicated in VKC, particularly in cases where secondary infections are involved [3,25]

The significance of *Staphylococcus* in ocular diseases, particularly in VKC extends beyond their presence on the ocular surface. This is mostly because of the chronicity of the disease, the opportunistic pathogenic nature of *Staphylococcus*, and its, ability to resist antimicrobial treatments by producing biofilms. It is not known as to whether the change in ocular surface conditions as in VKC has any effect on the bacterial phenotypes compared to those isolated from healthy individuals. Further, the emergence of multidrug-resistant strains complicates treatment options increasing the risk of therapeutic failure emphasing the need for continuous monitoring of bacterial resistance patterns [26]. Antimicrobial resistance (AMR) among the bacteria isolated from VKC is less studied in connection with the dynamics of bacterial diversity and ability to form biofilms, which is crucial for developing effective management strategies and improving patient care. Thus, this study aims to explore the bacterial diversity, prevalence, biofilm forming ability, and antimicrobial resistance in *Staphylococcus* species in VKC compared to healthy controls.

## 2 Materials and methods

### 2.1 Recruitment of subjects

This study was conducted as a case control study with the objective of investigating the burden of antimicrobial resistance in biofilm forming *Staphylococcus* spp. from the eyes of Individuals with Vernal Keratoconjunctivitis (VKC) compared to healthy individuals. Samples were collected over one year from February 2022 to February 2023 after the informed consent taken from the participants. The study was conducted at a single tertiary centre, LV Prasad Eye Institute Hyderabad. All the VKC participants were included in the study if they had both symptoms of allergy (itching, photophobia, redness, or ropy discharge) and had active allergy related ocular surface inflammation who have not been on any topical medications for at least 3 months prior to the presentation. The conjunctival swabs were collected from all the patients prior to initiation of any treatment. For this particular study severity scoring was not done but clinical assessment was done and based on clinical phenotype, they were categorized into palpebral (presence of diffuse conjunctival hyperemia and upper tarsal conjunctival papillae >1 mm), limbal (presence of atleast 1 clock hour of horner-tranta’s dots or thickening over the limbus) and mixed varieties (presence of both palpebral and limbal features).

Twenty six children with Vernal Keratoconjunctivitis (VKC) (20 males and 6 females), aged between 5 and 20 years (mean age 13.3 y) were recruited for the study. Additionally, twenty three children without ocular pathology (18 males and 5 females), ranging in age from 6 to 20 years (mean age 14.6 y), were enrolled in the study as healthy controls (HC) (Table 1). The exclusion criteria for the Vernal Keratoconjunctivitis group comprised individuals with other ocular or systemic diseases, and those who had used antibiotic drugs or eye drops in the past 2 months. The healthy control group includes individuals who had not undergone any ocular surgery or on medication for ocular or systemic diseases in the past 2 months. Before collecting samples, written informed consent was obtained from all study participants. The study was approved by the Institutional Review Board and complied with the declaration of the tenets of Helsinki.

**Table 1:** Demographic details of VKC and HC groups.

S. No	Parameter	Healthy control (HC)	Vernal Keratoconjunctivitis (VKC)	p value
1	Number of participants	23	26	NA
2	Age range	6–20	5–20	NA
	Group1 (5–10)	5	7	0.849
	Group2 (11–15)	6	8	0.888
	Group3 (16–20)	12	11	0.843
3	Average age	14.6	13.3	0.242
4	Male: Female	18:5	20:6	0.913
5	Category of VKC			
	Limbal (%)	0	50	NA
	Palpebral (%)	0	27	NA
	Mixed (%)	0	23	NA
6	Culture positive (%)	78.26	77	0.812
7	Previous treatment history	NA	Cromal forte eye drops used by 1 individual	NA
8	Number of antibiotic eye drop courses	NA	NONE	NA
9	Anti-allergic treatments	NA	NONE	NA
10	Corticosteroid use	NA	NONE	NA
11	Predisposing factors	NA	One participant had allergic dermatitis and is not on medication	NA
12	Infection type	NA	Bilateral	NA

NA = Not Applicable.

### 2.2 Sample collection

Conjunctival swab samples were collected from both the right and left eyes of individuals with Vernal Keratoconjunctivitis (VKC), and healthy controls. The sampling procedure involved gentle swabbing of the bulbar tarsal and forniceal conjunctiva of the lower lid after administering topical anaesthetic, proparacaine hydrochloride (0.5 %) eye drops. This process was performed six times for each eye and to facilitate the procedure, a sterile Isohelix swab (SK–1S; Isohelix, Harrietsham, Kent, United Kingdom) moistened with sterile phosphate-buffered saline (PBS) was used. Following collection, the conjunctival swabs were carefully transferred to sterile tubes containing 500 μl PBS (pH 7.4, Sigma-Aldrich, Bangalore, India). The collected samples were stored in −80 ^°^C until further analysed and unless mentioned. The samples were processed separately for each eye, without pooling, to ensure individual analysis of both the right and left eyes.

### 2.3 Isolation of bacteria from samples

Bacteria from the conjunctival samples were isolated by plating the samples on to Blood agar plate immediately after the collection. For this, a 50 μl sample was added to a blood agar plate and incubated under aerobic conditions. The inoculated plates were checked for growth until 96 h and lacking signs of growth after the 7th day of incubation were discarded. In instances of positive cultures, the resulting colonies were isolated, purified and their morphological characteristics were recorded. Subsequently, the unique purified colonies from each sample were identified by 16S rRNA gene sequencing.

### 2.4 DNA extraction, PCR amplification, sequencing

Genomic DNA extraction from bacterial isolates was accomplished using the QIAamp DNA minikit (Qiagen, Hilden, North Rhine-Westphalia, Germany), following the manufacturer's instructions. In the final step, DNA was eluted with 35 μl of AE buffer provided in the kit. The quality of the genomic DNA was assessed on agarose gel electrophoresis (agarose, 1 % w/v), and quantified using a Nanodrop spectrophotometer. (Thermo Fisher Scientific Inc., Waltham, Massachusetts, USA). Following the verification of DNA integrity, PCR amplification was carried out using primers 27F (5'GAGTTTGATCCTGGCTCAG 3') and 27R (5'ACGGCTACCTTGTTACGACTT 3'). The resulting PCR products were then sequenced by Sanger sequencing and identified by matching the sequence similarity with the nearest neighbour using, nucleotide BLAST (Basic Local Alignment Search Tool) search analysis.

### 2.5 Detection of biofilm formation

Biofilm formation in the bacterial isolates from both healthy and VKC cohorts was detected by the crystal violet method [24]. For this, the overnight grown bacterial culture was adjusted to 0.5 McFarland, then further diluted to 10,000 times with Brain Heart Infusion (BHI) medium, later 100 μl of this suspension was added to a 96-well plate containing 100 μl of BHI medium. The inoculated bacterial cultures were incubated at 37 °C for 72 h. Later, the broth was removed, and the wells were washed twice with 200 μl of phosphate-buffered saline (PBS) at pH 7.4. Subsequently, the wells were air-dried, and bacterial cells adhering to the wells were stained with 0.1 % (w/v) crystal violet. Following staining, each well of the plate was washed with 200 μl of PBS and air-dried. 200 μl of absolute ethanol was then added to extract the crystal violet and the concentration was quantified at 595 nm using a spectrophotometer (SpectraMax M3, Molecular Devices, San Jose, CA, USA). Wells without cells served as controls (optical density (OD) < 0.1 at 595 nm), and the OD values were subtracted from all the wells. Previously characterised biofilm-positive (OD > 0.3 at 595 nm) and biofilm-negative strains (OD < 0.3 at 595 nm) were used as controls for the study [24]. All the experiments were performed in triplicate to ensure reliability.

The biofilm-forming ability of isolates is assessed based on their optical density (OD) values from the crystal violet assay, compared to a calculated OD threshold (ODcut) [27]. The ODcut is determined by adding the average OD of the negative control to three times the standard deviation of the negative control OD values. Isolates with OD values less than or equal to ODcut are classified as non-biofilm formers. Those with OD values of 2, 4, or more than 4 times the ODcut are categorized as weak, moderate, and strong biofilm formers, respectively [27].

### 2.6 Evaluating the minimum inhibitory drug concentration

Bacterial isolates from the (VKC) group and the (HC) group were tested against 15 antibiotics. An overnight bacterial suspension in Brain Heart Infusion (BHI) broth was adjusted to a 0.5 McFarland standard and subsequently diluted 100-fold. A volume of 100 μl of this suspension was added to a 96-well plate containing 100 μl of an antibacterial agent at known concentrations. After preparing the desired concentrations of the drug the antimicrobial agents were diluted in a twofold manner, ranging from 64 μg/ml to 0.125 μg/ml. Each well contained the following controls: the growth control (the respective organism in BHI broth) and the sterility control (sterile BHI broth without bacteria). The antibiotic concentrations were added in descending order, from well 1 to well 10. The plates were then incubated at 37 °C for 18–24 h to determine the Minimum Inhibitory Concentration (MIC). After incubation, wells were examined for visible growth, and the MIC was determined as the lowest concentration of antimicrobial agent inhibited the growth of tested bacterial isolate.

### 2.7 Scanning electron microscopy

In this study, the temporal biofilm formation at time points 4, 24, 48, 72 and 96 h was studied for 3 bacterial species that showed high biofilm formation in VKC cohort and 3 similar bacterial species from healthy cohort by scanning electron microscopy. For this, the biofilm-positive cultures were grown on coverslips (Blue star, Mumbai, India) that were placed in a 12-well polystyrene plate (Nunclon™, Thermo Scientific, Roskilde, Denmark) for distinct time points namely 4, 24, 48, 72, and 96 h. Subsequently, the cover slips underwent three washes with autoclaved distilled water then subjected to fixation for a duration of 3 h using 250 μL of glutaraldehyde (2.5 % v/v). After the fixation step, the glass cover slips underwent another round of three washes with autoclaved distilled water. Dehydration was carried out over 20 min, progressing through graded ethanol concentrations (10 %, 25 %, 50 %, 70 %, 90 %, and 100 %, v/v). The final step involved allowing the cover slips to air dry overnight. For visualization purposes, the biofilms on the cover slips were subjected to gold sputtering for 60 s using a High Vacuum Evaporator (SC7620 PALARON Sputter Coater, Quorum Technologies Ltd., East Sussex, UK). The scanning electron microscope (SEM) (Carl Zeiss-Model EVO 18, Carl Zeiss, Germany) was utilized for observing the biofilms, with the voltage settings ranging between 5 and 20 kV.

### 2.8 Statistical analysis

Standard deviation (SD) and mean values were calculated for the continuous clinical data. While for the variables in categorical data generated for different samples such as relative abundance of bacterial phyla, genera and species are represented as percentage values. The data was analysed using Statistical Package for Social Sciences (SPSS, version 21.0) to calculate a p-value. P-value of <0.05 was considered statistically significant. Independent *t*-test was used to calculate the significant differences in the bacterial diversity between groups (https://www.graphpad.com/quickcalcs/ttest1/).

## 3 Results

### 3.1 Sample collection and clinical characteristics

A total of 49 individuals were recruited, comprising 23 healthy individuals and 26 vernal keratoconjunctivitis patients. The two groups were matched for age, gender, and ethnicity (Table 1). No significant difference in the mean age of VKC (13.3 y) and healthy (14.6 y) groups was observed. Male to female ratio (20:6) indicated male preponderance with VKC (Table 1). The participants from VKC group were categorized into palpebral (27 %), limbal (50 %) and mixed (23 %). Conjunctival swabs from all the participants were collected and used for bacterial isolation.

### 3.2 Bacterial diversity

**Fig. 1:**
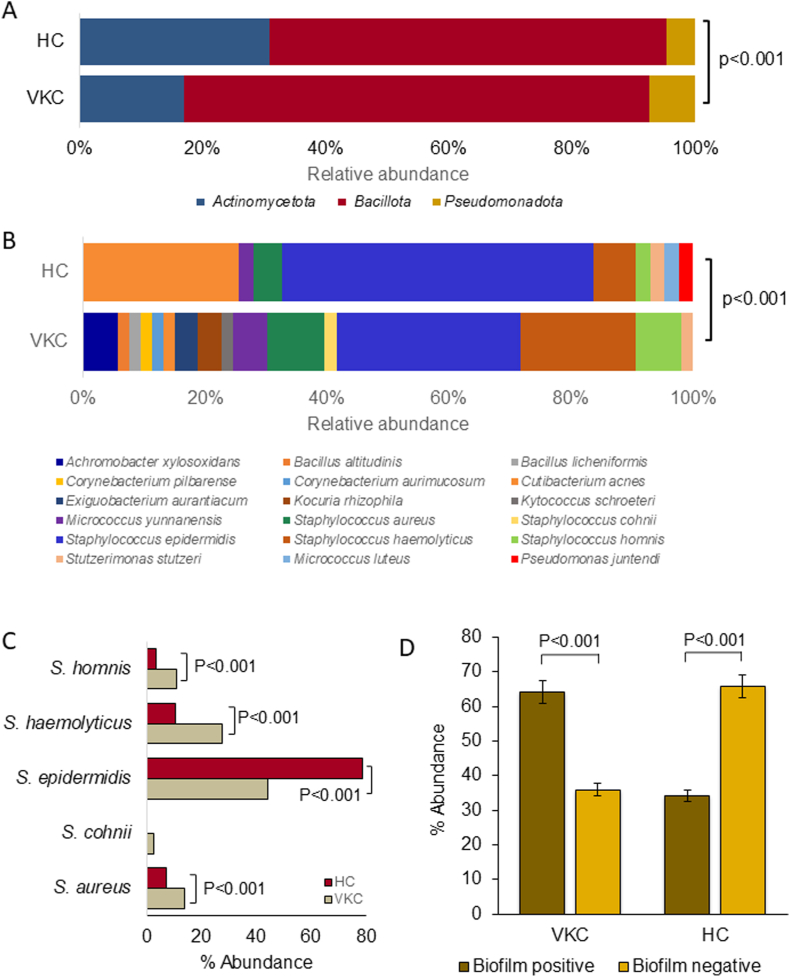
Bacterial diversity in allergic conjunctivitis and healthy control group. A. Relative abundance of bacterial Phyla in HC and VKC Group. B. Relative abundance of bacterial genera in HC and VKC Group. C. Relative abundance of *Staphylococcus* species. D. Percentage of biofilm forming bacteria in HC and VKC Group.

Among the total participants, 77 % (20) of the samples from VKC patients demonstrated culture positivity while 78.26 % (18) samples from HC group were culture positive. A total of 95 bacteria were isolated from all the samples and were identified by 16S rRNA gene sequencing followed by identification the nearest neighbour based on sequence similarity(Tables S1A and B). Further on the number of bacteria isolated from VKC and HC groups, in the VKC group, out of 26 individuals, 11.53 % had monospecies bacterial isolates, 38.46 % individuals displayed 2 bacterial species, 15.38 % had three species, 11.53 % had bacterial species ≥3 and 23.07 % did not show any growth. In the HC group, out of 23 individuals, 13.4 % had monospecies bacteria, 39.13 % individuals displayed dual species isolation, 17.39 % individuals had three bacterial species, 8.69 % individuals had ≥3 species, while 21.73 % of them did not show any growth. Though the overall mono, dual and three bacterial isolation from both the cohorts is not different, population having >3 bacterial species are significantly high in VKC (11.53 %) compared to HC (8.69 %). Three prominent phyla such as *Pseudomonadota, Bacillota* and *Actinomycetota* were significantly different in VKC group compared to HC (Fig. 1A). Sixteen bacterial species were identified in the VKC group that include *Achromobacter xylosoxidans* (5.6 %)*, Bacillus altitudinis* (1.9 %)*, Bacillus licheniformis* (1.9 %)*, Corynebacterium pilbarense* (1.9 %)*, Corynebacterium aurimucosum* (1.9 %)*, Cutibacterium acnes* (1.9 %)*, Exiguobacterium aurantiacum* (3.8 %)*, Kocuria rhizophila* (3.8 %)*, Kytococcus schroeteri* (1.9 %)*, Micrococcus yunnanensis* (5.6 %)*, Staphylococcus aureus* (9.4 %)*, Saphylococcus cohnii* (1.9 %)*, Staphylococcus epidermidis* (30.2 %)*, Staphylococcus haemolyticus* (18.9 %)*, Staphylococcus hominis* (7.5 %)*,* and *Stutzerimonas stutzeri* (1.9 %) (Fig. 1B). On the other hand in (HC group) samples nine species were observed that include *Cutibacterium acnes* (26.19 %)*, Micrococcus luteus* (2.38 %)*, Micrococcus yunnanensis* (2.38 %)*, Pseudomonas juntendi* (2.38 %)*, Staphylococcus aureus* (2.38 %)*, Staphylococcus epidermidis* (52.38 %)*, Staphylococcus haemolyticus* (7.14 %)*, Staphylococcus hominis* (2.38 %) and *Stutzerimonas stutzeri* (2.38 %). Significant change in bacterial diversity in VKC compared to HC was observed (Table 2; Fig. 1A and B). The bacterial species such as *Staphylococcus epidermidis* (52.38 % abundance) and *Cutibacterium acnes* (26.19 %) are abundant in healthy group, while in VKC group, bacterial species such as *Staphylococcus epidermidis* (30.2 %) and *Staphylococcus haemolyticus* (18.9 %) are abundant (Fig. 1C). In VKC group the abundance of *Cutibacterium acnes* was 1.9 % while in healthy control it was 26.19 %. Overall, the abundance of *Staphylococcus* spp. is high in both VKC and healthy with 67.92 %–64.28 % abundance respectively. Genera such as *Achromobacter* (5.66 %), *Bacillus* (3.77 %), *Corynebacterium* (3.77 %), *Exiguobacterium* (3.77 %), *Kokuria* (3.77 %) and *Kytococcus* (1.89 %) were observed in VKC group only.

**Table 2:** Comparison of percentage abundance of isolated bacteria between HC and VKC Groups.

Bacterial genera	Percentage Abundance in HC	Percentage Abundance in VKC	p value
*Cutibacterium*	26.19	1.9	0.001
*Micrococcus*	4.76	5.66	0.555
*Pseudomonas*	2.38	0	0.555
*Staphylococcus*	64.28	67.92	0.001
*Stuzerimonas*	2.38	1.9	1.000
*Achromobacter*	0	5.66	0.079
*Bacillus*	0	3.77	0.240
*Corynebacterium*	0	3.77	0.240
*Exiguobacterium*	0	3.77	0.240
*Kocuria*	0	3.77	0.240
*Kytococcus*	0	1.89	0.555

Changes in the bacterial diversity was assessed for different age groups and the type of VKC such as Limbal, Palpebral and Mixed. The bacterial diversity among the 3 different age groups of HC indicated that *Staphylococcus* was predominantly present in all age groups ranging from 58.8 % to 100 % of abundance (Table S2A). On the other hand, VKC had more bacterial diversity in all age groups with predominance of *Staphylococcus* ranging from 58 % to 75 % (Table S2A). Genus *Cutibacterium* emerged was the second predominant bacterium in HC with 42.3 % of the total abundance in Group3 that covers children above 16 years, however in VKC no such presence was noted. Among the different types of VKC, Limbal variety had *Staphylococcus haemolyticus* (30.4 %), *Staphylococcus epidermidis* (13 %) and *Achromobacter xylosoxidans* (13 %) as major bacteria (Table S2B). Palpebral variety had *Staphylococcus epidermidis* (57.4 %) and Mixed variety had *Staphylococcus epidermidis* (38.9 %) *Staphylococcus haemolyticus* (16.7 %) and *Staphylococcus aureus* (16.7 %) as predominant bacteria (Table S2B). The phylogenetic analysis using 16S rRNA gene sequencing did not show clear differences among the VKC and HC bacterial isolates (Fig. S1).

#### 3.2.1 Biofilm formation by the bacterial isolates

The bacterial isolates from both the HC and VKC groups were screened for their ability to form biofilm. Among the 53 bacterial isolates from VKC group 34 (64.15 %) could form biofilms while in HC group 13 out of 42 bacterial isolates (31 %) showed bio-film forming ability (p < 0.001) (Fig. 1D). Biofilm-forming ability of Bacterial isolates is categorized based on their optical density (OD) values of the crystal violet dye extract relative to a calculated threshold (ODcut) [27]. The criteria for the ODcut is calculated from the OD average of negative control. When the OD of the negative control is equal to the OD of the test culture, it is considered a non-biofilm-former. When the OD is 2, 3 or 4 times the negative control it was considered a weak, moderate and strong biofilm-former respectively. The major biofilm producers in VKC group include *Staphylococcus aureus, Staphylococcus epidermidis, Staphylococcus haemolyticus,* and *Staphylococcus hominis*. In HC group, bacteria such as *Cutibacterium acnes, Staphylococcus epidermidis* indicated higher biofilm formation.

#### 3.2.2 Scanning electron microscopic imaging of biofilm formation

**Fig. 2:**
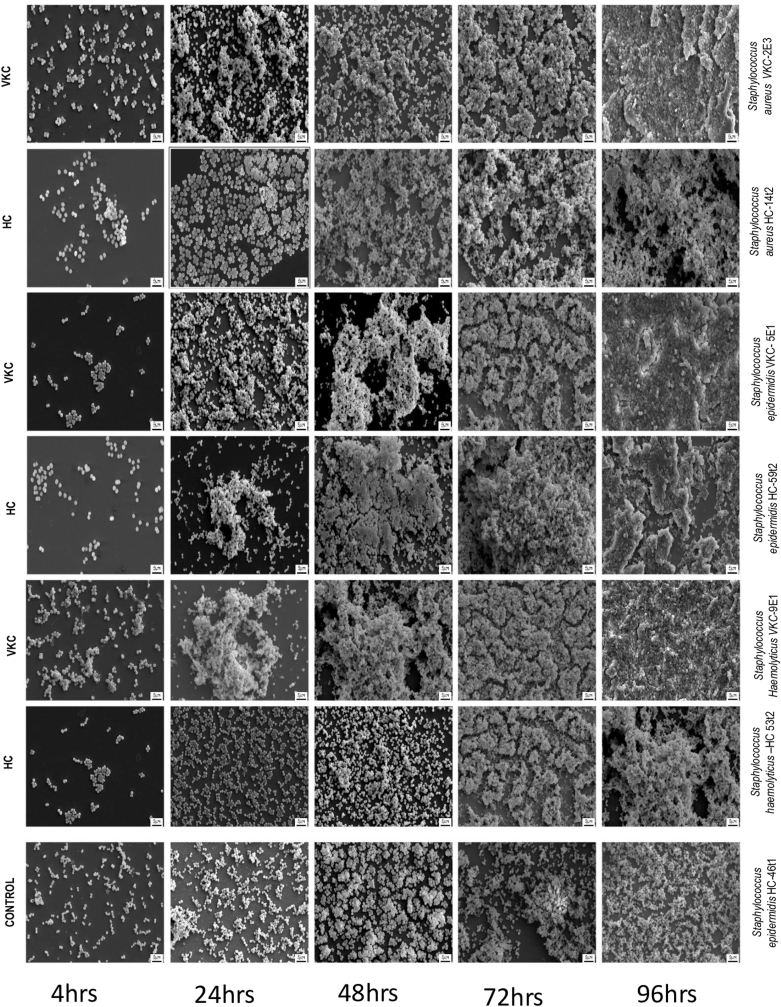
Temporal biofilm formation in *Staphylococcus* species visualized by Scanning electron microscopy. VKC= Isolate from vernal keratoconjunctivitis; HC= Isolate from healthy individual.

Temporal formation of biofilm at different time intervals i.e 4h, 24h, 48h, 72h, and 96h was imaged using scanning electron microscope (SEM). The top biofilm forming bacterial isolates from VKC group i.e., *Staphylococcus aureus* VKC-2E3*, Staphylococcus epidermidis* VKC-5E1, and *Staphylococcus haemolyticus* VKC-9E1 and *Staphylococcus aureus* HC-14t2*, Staphylococcus epidermidis* HC-59t2, and *Staphylococcus haemolyticus* HC-53t2 from HC group were selected and compared the biofilm formation (Fig. 2). SEM analysis revealed a substantial disparity in biofilm formation between strains in the VKC cohort compared to those in the healthy group at various time intervals i.e. 4h, 24h, 48h and 72h. SEM images indicated that all *Staphylococcus* isolates from VKC group showed early formation of biofilm compared to healthy. By 24h the isolates in VKC cohort showed multi layered aggregation of cells. However, by 48h the isolates from both cohorts showed aggregation of the cells. At 96h no particular difference in the biofilm formation was observed among the isolates of both cohorts. A non-biofilm forming isolate *Staphylococcus epidermidis* HC- 46t1 served as the control.

### 3.3 Minimum inhibitory concentrations of biofilm forming bacteria

The MIC was tested for the biofilm forming bacteria from both VKC and healthy groups (Tables S3A and B). Total of 15 antibiotics were used to determine the MIC in 27 bacterial isolates from the VKC group and 15 isolates from the HC group. Notably all isolates from the VKC group exhibited resistance to multiple antibiotics (>2antibiotics) compared to the HC group (Table 3A). Out of 27 isolates from the VKC group, 21 isolates demonstrated resistance to more than two antibiotics highlighting the prevalence of Multiple antibiotic resistance strains (Table S3A and Table 3B). Among the *Staphylococcus* species tested for antibiotic susceptibility from VKC group, 80 % of them exhibited multidrug resistance, while in HC 33 % of them were multi-drug resistance (MDR) (Table 3B). In HC group among the 15 isolates, 9 showed resistance to more than two antibiotics, and 3 isolates exhibited resistance to only a single antibiotic (Table S3B and Table 3B). Table 3A and B shows the percentage of *Staphylococcus* spp. sensitive and resistance to the tested antibiotics in both VKC and HC groups. All *Staphylococcus* species in HC group exhibited 100 % sensitivity to antibiotic vancomycin, while in VKC group 6.6 % showed resistance. All the tested Fluoroquinolones such as ciprofloxacin, moxifloxacin and ofloxacin were sensitive to 100 % of *Staphylococcus* spp. in HC group. While in VKC group, Fluoroquinolones exhibited resistance in 46–60 % of the *Staphylococcus* species. Additionally, 73 % of the isolates in VKC group exhibited resistance to clindamycin. When tested for the susceptibility patterns for Fusidic acid, one of the common ocular antibiotic routinely used for ocular infections, it demonstrated 60 % susceptibility and 40 % resistance among the *Staphylococcus* bacteria in the VKC group, whereas in the HC group, it showed susceptibility for 77.7 % of the *Staphylococcus* spp. and resistance to 22.2 %. Interestingly, Gatifloxacin that is routinely used in India, for ocular infections, exhibited 100 % susceptibility for all *Staphylococcus* species in both the VKC and HC groups. These findings indicates that the strains from the VKC group showed higher resistance compared to healthy.

**Table 3A:** Percentage of resistance among *Staphylococcus* species to antibiotics in both HC and VKC groups.

Antibiotic class	Antimicrobial agent	VKC group			HC group			P value∗
		MIC range	S%	R%	MIC range	S %	R%	
Aminoglycosides	Gentamicin	0.25 - ≥64	26.6	73.3	0.125–64	66.6	33.3	0.001
	Amikacin	2.0- ≥64	73.3	26.6	2.0–64	88.8	11.1	0.079
	Tobramycin	0.5 - ≥ 64	33.3	66.6	0.125–64	66.6	33.3	0.001
	Streptomycin	2.0–64	NA	NA	0.125–64	NA	NA	NA
Fluoroquinolones	Ciprofloxacin	0.125–64	46.6	53.3	0.125–0.25	100	0	0.001
	Moxifloxacin	0.125–8	60	40	0.125	100	0	0.001
	Ofloxacin	0.125–32	53.3	46.6	0.125–2	100	0	0.001
	Gatifloxacin	0.03–1	100	0	0.078–1	100	0	1.000
Penicillin	Ampicillin	0.125- ≥64	26.6	73.3	0.125–16	44.4	55.5	0.001
Lincosamide	Clindamycin	1 - ≥64	26.6	73.3	0.125–64	55.5	44.4	0.001
Cephalosporin	Cefuroxime	0.25-≥64	60	40	0.125–64	66.6	33.3	0.264
	Ceftazidime	0.5-≥64	60	40	0.5–64	55.5	44.4	0.470
	Cefazolin	0.125-≥64	80	20	0.125–64	66.6	33.3	0.168
Macrolides	Azithromycin	0.125–64	46.6	53.3	0.125–64	33.3	66.6	0.299
Glycopeptide	Vancomycin	0.5–64	93.3	6.6	0.125–8	100	0	0.020
Corticosteroids	Triamcinolone	>64	NA	NA	>64	NA	NA	NA
Fusidane	Fusidic Acid	0.125–16	60	40	0.125–4	77.7	22.2	0.001

MIC = minimum inhibitory concentration, S = sensitive, R = resistance, NA = not available; ∗ = p value calculated for resistance percentage values of HC and VKC group.

**Table 3B:** Antimicrobial susceptibility and multidrug resistance in *Staphylococcus* species from VKC and HC groups.

Bacterial isolates	Sample	Biofilm potential	Aminoglycosides			Fluoroquinolones				Penicillin	Lincosamide	Cephalosporin			Fusidane	Macrolides	Glycopeptide	MDR
			Gentamicin	Amikacin	Tobramycin	Ciprofloxacin	Moxifloxacin	Ofloxacin	Gatifloxacin	Ampicillin	Clindamycin	Cefuroxime	Ceftazidime	Cefazolin	Fusidic acid	Azithromycin	Vancomycin	
*S.haemolyticus*	VKC-21E1	9.25	64(R)	8(S)	>64 (R)	4(R)	0.5(S)	4(R)	0.06 (S)	0.5(R)	>64(R)	8(S)	32(R)	2(S)	0.5(S)	1(S)	2(S)	MDR
*S.cohnii*	VKC-7E1	6.66	32(R)	4(S)	64(R)	8(R)	0.5(S)	16(R)	0.06 (S)	16(R)	32(R)	>64(R)	>64(R)	32(R)	1 (R)	>64(R)	4(S)	MDR
*S.haemolyticus*	VKC-9E1	12.33	64(R)	8(S)	>64(R)	32(R)	4(R)	32(R)	1 (S)	2(R)	32(R)	8(S)	32(R)	2(S)	16 (R)	>64(R)	2(S)	MDR
*S.hominis*	VKC-6AT1	1.15	1(S)	2(S)	1(S)	0.25(S)	0.125(S)	0.5(S)	0.03 (S)	0.125(S)	16(R)	0.5(S)	2(S)	0.5(S)	8 (R)	0.5(S)	0.5(S)	NO
*S.homnis*	VKC-6AT4	2.15	>64(R)	64(R)	>64(R)	1(S)	0.125(S)	1(S)	0.03 (S)	4(R)	>64(R)	32(R)	64(R)	4(S)	8 (R)	>64(R)	4(S)	MDR
*S.epidermidis*	VKC-8E1	2.00	16(R)	4(S)	1(S)	0.5(S)	0.125(S)	0.5(S)	0.03 (S)	0.125(S)	8(R)	2(S)	2(S)	0.125(S)	0.5(S)	0.125(S)	1(S)	NO
*S.hominis*	VKC-9AT	12.39	0.25(S)	4(S)	2(S)	0.125(S)	0.125(S)	0.5(S)	0.03 (S)	0.5(R)	2(S)	8(S)	8(S)	0.25(S)	0.5 (S)	1(S)	1(S)	NO
*S.epidermidis*	VKC-16AT1	2.00	64(R)	16(R)	>64(R)	0.125(S)	0.125(S)	0.5(S)	0.03 (S)	2(R)	1(S)	4(S)	8(S)	1(S)	0.5 (S)	>64(R)	4(S)	MDR
*S.epidermidis*	VKC-12E1	1.96	>64(R)	64(R)	>64(R)	1(S)	4(R)	4(R)	0.03 (S)	2(R)	>64(R)	32(R)	64(R)	2(S)	0.125 (S)	64(R)	16(S)	MDR
*S.haemolyticus*	VKC-6AT2	4.26	0.25(S)	2(S)	0.5(S)	0.125(S)	0.125(S)	0.125(S)	0.03 (S)	1(R)	>64(R)	32(R)	4(S)	4(S)	16(R)	1(S)	1(S)	MDR
*S.haemolyticus*	VKC-14E2	2.01	32(R)	4(S)	>64(R)	4(R)	0.125(S)	2(S)	0.06 (S)	0.25(S)	>64(R)	4(S)	16(S)	2(S)	0.125 (S)	1(S)	4(S)	MDR
*S.aureus*	VKC-22E2	3.27	16(R)	8(S)	2(S)	64(R)	4(R)	32(R)	0.03 (S)	0.5(R)	1(S)	0.25(S)	4(S)	0.125(S)	0.125 (S)	>64(R)	2(S)	MDR
*S.aureus*	VKC-2E3	4.20	1(S)	4(S)	4(R)	32(R)	2(R)	16(R)	1 (S)	8(R)	2(S)	64(R)	>64(R)	32(R)	0.125 (S)	>64(R)	4(S)	MDR
*S.aureus*	VKC-22E1	2.01	32(R)	>64(R)	32(R)	64(R)	8(R)	32(R)	0.03(S)	0.125(S)	16(R)	4(S)	4(S)	0.25(S)	0.125 (S)	16(R)	4(S)	MDR
*S.aureus*	VKC-2AT1	2.23	4(R)	8(S)	8(R)	2(R)	8(R)	2(S)	0.06 (S)	>64(R)	>64(R)	>64(R)	0.5(S)	>64(R)	1(R)	2(S)	>64(R)	MDR
*S.epidermidis*	HC-49t2	0.79	0.125(S)	4(S)	1(S)	0.125(S)	0.125(S)	0.125(S)	0.078 (S)	0.125(S)	0.125(S)	0.125(S)	0.5(S)	0.125(S)	0.125 (S)	0.125(S)	0.125(S)	NO
*S.epidermidis*	HC-46t1	1.94	2(S)	8(S)	2(S)	0.25(S)	0.125(S)	0.25(S)	0.078 (S)	0.25(S)	2(S)	1(S)	16(S)	0.25(S)	0.5 (S)	2(S)	1(S)	NO
*S.haemolyticus*	HC-53t1	4.31	8(I)	4(S)	16(R)	0.5(S)	0.25(S)	2(S)	1 (S)	16(R)	64(R)	>64(R)	>64(R)	>64(R)	4 (R)	64(R)	4(S)	MDR
*S.haemolyticus*	HC-53t2	1.53	8(I)	2(S)	16(R)	0.5(S)	0.125(S)	0.5(S)	1 (S)	16(R)	64(R)	>64(R)	>64(R)	>64(R)	4 (R)	64(R)	4(S)	MDR
*S.epidermidis*	HC-46t2	1.94	4(S)	16(S)	0.125(S)	0.5(S)	0.125(S)	1(S)	0.03 (S)	8(R)	32(R)	16(S)	64(R)	4(S)	0.5 (S)	64(R)	8(S)	NO
*S.epidermidis*	HC-18t2	2.93	32(R)	2(S)	2(S)	0.5(S)	0.125(S)	0.5(S)	0.03 (S)	0.25(S)	2(S)	0.125(S)	4(S)	0.25(S)	0.125 (S)	>64(R)	0.5(S)	NO
*S.epidermidis*	HC-13t1	4.14	>64(R)	>64(R)	>64(R)	0.25(S)	0.125(S)	0.5(S)	0.078 (S)	8(R)	1(S)	16(S)	64(R)	4(S)	0.125 (S)	64(R)	4(S)	MDR
*S.epidermidis*	HC-59t2	0.90	4(S)	4(S)	8(S)	0.25(S)	0.125(S)	2(S)	0.03 (S)	16(R)	64(R)	>64(R)	64(R)	64(R)	0.125 (S)	1(S)	2(S)	NO
*S.epidermidis*	HC-50t3	2.23	2(S)	4(S)	2(S)	0.25(S)	0.125(S)	0.5(S)	0.078 (S)	0.125(S)	1(S)	0.5(S)	4(S)	0.125(S)	0.125 (S)	64(R)	4(S)	NO

S = sensitive; R = resistance; + = Biofilm positive; - = Biofilm negative; MDR = multi drug resistance; NO = Absence of MDR.

Note: Bacterial strain is classified as Multi Drug Resistant (MDR) if it exhibits resistance to at least one antibiotic in three different classes. In the VKC group out of 15 strains tested 11 demonstrated multi drug resistance. These strains showed resistance to at least three different classes of drugs including Aminoglycosides, Fluoroquinolones, Cephalosporins, Macrolides, Penicillins, and Lincosamides. In the healthy group out of 9 strains tested 3 strains exhibited resistance to aminoglycosides, cephalosporins, and macrolides, so these isolates are considered as MDR strains.

#### 3.3.1 Correlating the biofilm forming ability with the multiple antibiotic resistance

**Fig. 3:**
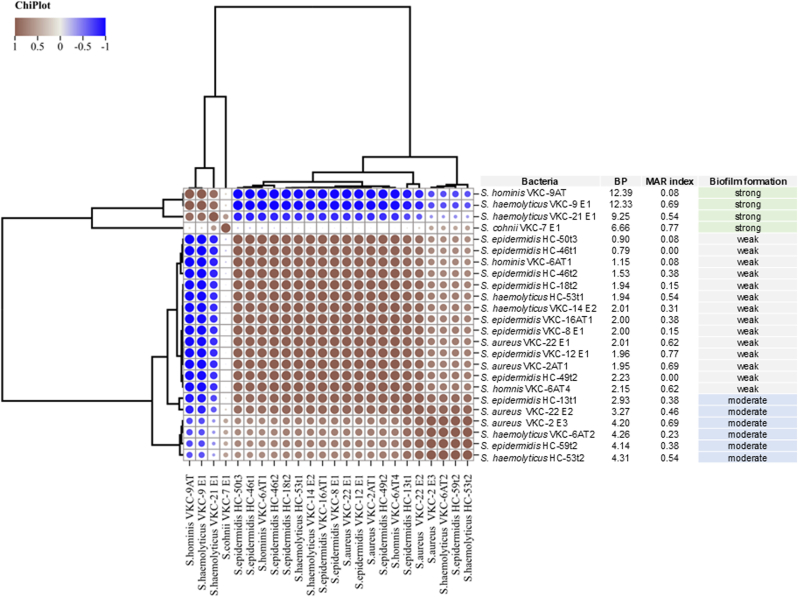
Correlation of biofilm potential and multiple antibacterial resistance in *Staphylococcus* species.

The biofilm forming potential (BP) and the Multiple Antibiotic Resistance (MAR) index of the *Staphylococcus* species from VKC and HC groups were measured and correlated [28,29]. The biofilm forming potential (BP) was calculated from the values of Biofilm Formation Index (BFI). The BFI was used to quantify the biofilm-forming ability of bacterial isolates by comparing the biofilm biomass that is measured by crystal violet assay and from the cell density. It is calculated using the formula, BFI=(B–C)/G. In this formula, B is the absorbance of the crystal violet, C is the absorbance of the control wells and (without biofilm formation) and G is the absorbance of the cell density in the broth. From the BFI values, BP was calculated as 1−BFI sample average/BFI of negative control [28]. The BP and MAR were measured for all the *Staphylococcus* species that are biofilm positive. A positive correlation for the biofilm forming strength of the bacterial isolates with the MAR index was observed (Fig. 3). The correlation graph showed discrete clusters for the strong, medium and weak biofilm producers.

## 4 Discussion

Vernal keratoconjunctivitis (VKC) is a severe form of ocular allergy mostly prevalent in hot and arid geographical conditions such as Indian subcontinent and Africa. VKC is a severe form of ocular allergy, most commonly observed in hot and arid regions such as the Indian subcontinent and Africa. Interestingly, VKC is also prevalent in countries with temperate climates, including Italy [30] VKC is typically seasonal affecting mostly male children and young adults [31,32]. Our data also indicated male children preponderance with VKC (Table 1). Although, in most cases of VKC, the signs and symptoms are resolved naturally by the onset of puberty, longitudinal studies have shown that the eyes may be worsened in 2.7 % of the cases affecting their vision [31]. In the recent past the role of ocular surface bacteria in VKC has gained attention and the studies have shown increase in the bacterial diversity in VKC patients and involvement in secondary infections [33,34]. Culture positivity up to 77.8 % from the allergic conjunctivitis ocular samples was reported earlier [14,16]. In the present study, though the overall bacterial counts are less in VKC compared to HC, the VKC group exhibited a significantly high bacterial diversity compared to healthy controls (HC) with no significant difference in culture positivity (Fig. 1A and B; Table 1). VKC group had significantly high abundance of the opportunistic pathogens such as *Staphylococcus, Corynebacterium, Exiguobacterium* and *Micrococcus*, while *Cutibacterium*, which is a skin and ocular surface commensal decreased in abundance. The increased bacterial diversity in the VKC group suggests an association with more complex and potentially pathogenic microbial environment [35]. Despite a similar proportions of bacterial abundance is shared between the male and females of HC and VKC groups, their biofilm forming ability and antimicrobial susceptibilities are different. Both male and female isolates of HC had no strong biofilm forming bacteria, while in VKC, 7.1 % and 20.5 % isolates from female and males respectively were strong biofilm producers. Antimicrobial resistance among the bacterial isolates from both male and female remained same with 43 %, while the isolates from males in HC had higher resistance (36 %) compared to isolates from females (23 %) (Tables S4A–C). Such gender disparity in the antimicrobial resistance was earlier reported in uropathogenic *E. coli* indicating this as common phenomena [36].

The results of the study also indicated high abundance of the genus *Staphylococcus* compared to other bacterial genera in both VKC (67.92 %) and HC (64.28 %) groups (Table 2). Genus *Staphylococcus* includes diverse bacterial species that are commensal to the skin and ocular surface [24]. Several studies have highlighted that *Staphylococcus* spp. are the most common causative agent identified from ocular infections [37,38]. Significant difference in the abundance among the four *Staphylococcus* species such as *S. aureus, S. epidermidis, S. haemolyticus* and *S. hominis* is observed in VKC compared to HC group (Fig. 1C). Notably, genus *Staphylococcus* has been characterised as a resident to human body and are connected with the biofilm associated infections that are difficult to treat [24,39,40]. Thus, the biofilm forming ability was assessed for all the bacteria isolated from VKC and HC. The number of bacteria that formed biofilm in VKC (64.15 %) are significantly higher than the HC group (31 %) (Fig. 1D). This observation is in line with earlier studies where the biofilm formation was linked to the increased pathogenicity and chronic infection [[41], [42], [43]]. Further, *S. aureus, S. epidermidis, S. haemolyticus* that showed high biofilm forming ability in VKC were used to understand the temporal biofilm formation by the scanning electron microscopy (SEM) (Fig. 2). Significant increase in the biofilm forming ability in VKC group is an indication that the isolates of VKC group particularly of the genus *Staphylococcus* has an association with the prevailing chronic disease condition. It has been reported earlier that *Staphylococcus* species from patient samples have more biofilm forming ability compared to the isolates from healthy individuals [44]. Further, the biofilms are more resistant to antimicrobial agents and the immune system [45,46]. Thus, the bacterial isolates from VKC and healthy groups were screened for their antimicrobial susceptibility to 15 antibiotics. The results indicated high proportion of the Staphylococcal isolates from VKC group (80 %) were resistant to more than 2 antibiotics, comparatively, in HC the numbers are less (33 %) (Table 3B). Fluoroquinolones are the most frequently used first line antibiotics for ocular infections [47]. While, *Staphylococcus* spp. from VKC showed resistant to all the 3 fluoroquinolones in >40 % of the isolates. Whereas the isolates from the HC group were 100 % susceptible to all the 3 fluoroquinolones tested. Notably, a high percentage of *Staphylococcus* species exhibited resistance to Clindamycin in VKC group (73.3 %) and in the HC group (44.4 %). Studies on endophthalmitis showed that 20 % of the isolates of Coagulase-negative Bacteria (CoNB) and *S. aureus* exhibited resistance to Clindamycin [48]. The resistance trends of ocular *S. aureus* isolates from 2015 to 2019 indicate an increase in the resistance to clindamycin from 44.4 to 50 %, indicating rise in the resistance to these bacteria [37]. *S. aures* VKC-2AT1 strain from VKC group exhibited resistance to the maximum vancomycin concentration i.e., 64 μg/ml tested in the study. The high antibiotic resistance in the biofilm forming bacteria of VKC group indicates that these strains might be more challenging to treat and manage [49,50]. Further, a positive correlation between the strength of the biofilm formation and multiple antibiotic resistance was established (Fig. 3). Although it is generally accepted that the antimicrobial susceptibility decreases with the biofilm formation, the biofilm associated antimicrobial susceptibility tests in clinical samples is not a common practice [51]. Thus, it would be appropriate to screen for antibiotic resistance and biofilm formation of the causative bacteria and use suitable antibiotics in VKC patients with secondary infections. The results suggest the need for the screening of antimicrobial resistance profiles in VKC bacterial isolates from time to time to understand the resistance pattern. Further, bacteria that were isolated in VKC patients with more than three species demonstrated the highest proportion of moderate to strong biofilm formation (61 %) and 71.43 % of them exhibited multi drug resistance. These findings indicate that polymicrobial infections particularly those involving multi species, are more likely to produce strong biofilms and exhibit significant antimicrobial resistance.

Despite the fact that this study provides valuable insights, the limitations include low sample size and lack of ethnic diversity may limit the generalizability of the study findings. Further, longitudinal studies may help in understanding the temporal dynamics of microbial changes and antimicrobial resistance in vernal keratoconjunctivitis patients.

## 5 Conclusions

In conclusion, the results emphasize that the VKC is not only associated with increase in bacterial diversity but also shelters diverse biofilm forming *Staphylococcus* species. This study raises concerns over the high prevalence of antimicrobial resistance in *Staphylococcus* species isolated from VKC patients compared to those from healthy controls. These findings highlight the critical importance of evaluating biofilm formation and antimicrobial resistance in ocular isolates from VKC patients to ensure the selection of effective treatment strategies, thereby minimizing the risk of therapeutic failure and reducing the chances of morbidity and corneal complications.

## CRediT authorship contribution statement

**Nelaveni Rupa:** Writing – original draft, Methodology, Formal analysis. **Pragnya Rao Donthineni:** Writing – review & editing, Resources, Project administration, Investigation. **Sayan Basu:** Resources, Project administration, Funding acquisition. **Kotakonda Arunasri:** Writing – review & editing, Supervision, Methodology, Investigation, Data curation, Conceptualization.

## Data availability statement

All the data required for replicating the study is provided in the main text as well as in supplementary information.

## Funding

This research was funded by Institutional grant (20234).

## Competing interest statement

"I have nothing to declare".

## Data Availability

No data was used for the research described in the article.
