# Supplementary material for: The burden of antimicrobial resistance in biofilm forming Staphylococcus spp. from Vernal Keratoconjunctivitis patients eyes
Source: Biofilm. 2025 Apr 5;9:100278. doi: 10.1016/j.bioflm.2025.100278 (PMC12018567; doi:10.1016/j.bioflm.2025.100278)
Supplement: Multimedia component 1 [file mmc1.docx]

**Supplementary Data**

**
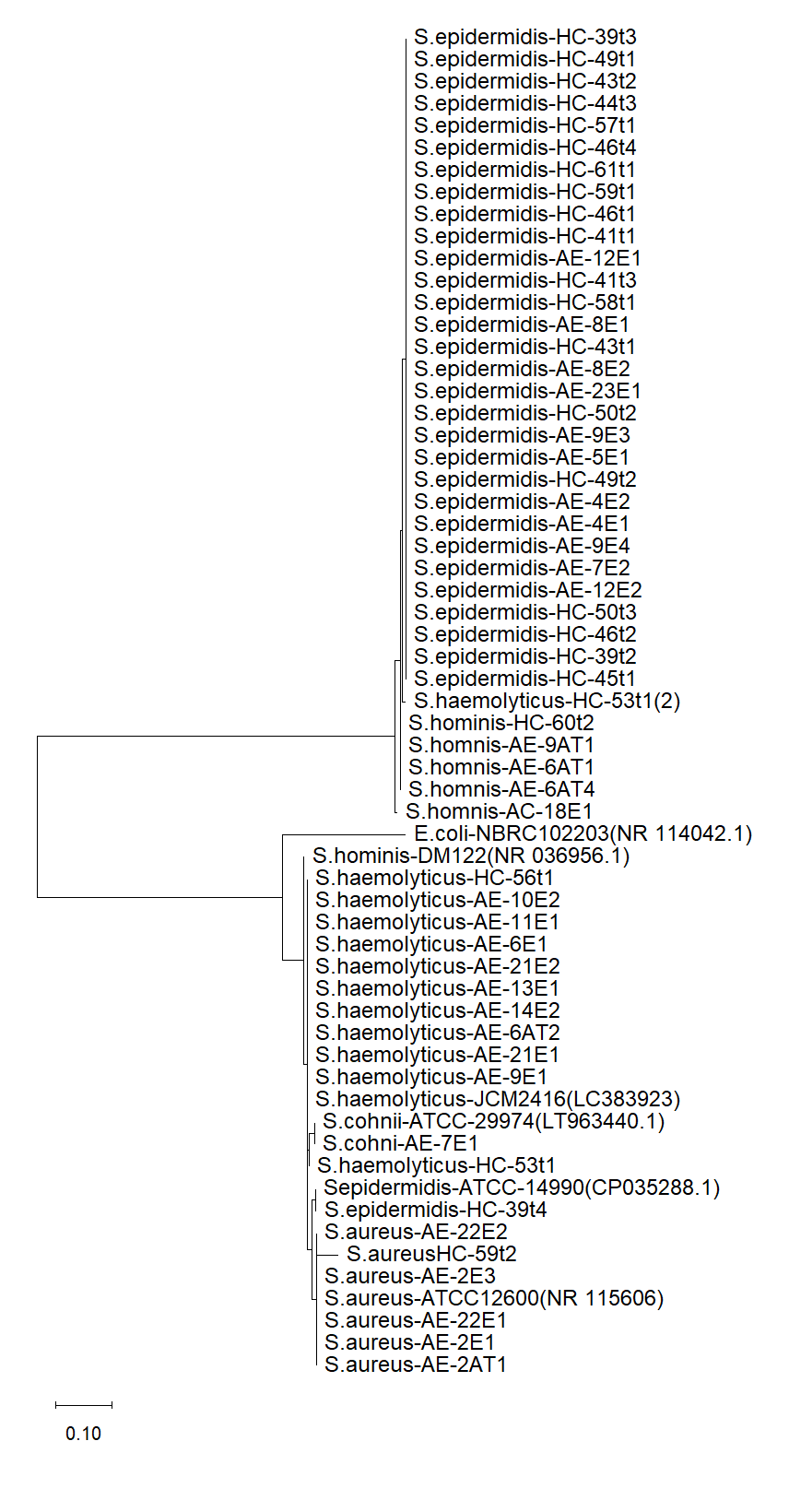
**

Fig S1. Phylogenetic tree was computed using the Neighbor-joining tree method for the 16S rRNA gene sequences of Staphylococcus species along with their respective Type strains. Bootstrap values after 500 resamplings (in %) are given at the nodes. *Escherichia coli* NBRC 102203 (NR 114042.1) is used as out tree.

Table 1: BLAST analysis results of the VKC isolates' nearest neighbors in the database and their percentage relatedness.

| CODE | NEAREST_NEIGHBOUR | ACCESSION_NUMBER | SIMILARITY (%) | QUERY COVERAGE (%) |
| --- | --- | --- | --- | --- |
| VKC_AE1 | *Bacillus licheniformis* | CP093290.1 | 99.4 | 100 |
| VKC_2 E1 | *Staphylococcus aureus* | CP040998.1 | 97.58 | 100 |
| VKC_2 E2 | *Kocuria rhizophila* | NR_026452.1 | 99 | 100 |
| VKC_2 E3 | *Staphylococcus aureus* | CP011526.1 | 99 | 100 |
| VKC_4 E1 | *Staphylococcus epidermidis* | [CP035288.1](https://www.ncbi.nlm.nih.gov/nucleotide/CP035288.1?report=genbank&log$=nucltop&blast_rank=3&RID=VSH925FP016) | 99.89 | 100 |
| VKC_4 E2 | *Staphylococcus epidermidis* | LN681574.1 | 99.89 | 100 |
| VKC_ 5E1 | *Staphylococcus epidermidis* | KT989845.1 | 99.72 | 100 |
| VKC_5E2 | *Micrococcus yunnanensis* | NR_116578.1 | 98.78 | 100 |
| VKC_5 E3 | *Stutzerimonas stutzeri* | [NR_041715.1](https://www.ncbi.nlm.nih.gov/nucleotide/NR_041715.1?report=genbank&log$=nucltop&blast_rank=1&RID=VUH4X8DM013) | 96.76 | 100 |
| VKC_6 E1 | *Staphylococcus haemolyticus* | CP035291.1 | 98.39 | 100 |
| VKC_6 E2 | *Micrococcus yunnanensis* | [NR_116578.1](https://www.ncbi.nlm.nih.gov/nucleotide/NR_116578.1?report=genbank&log$=nucltop&blast_rank=11&RID=VUHGPC4K013) | 98.55 | 100 |
| VKC_6 E3 | *Kocuria rhizophila* | [KJ476722.1](https://www.ncbi.nlm.nih.gov/nucleotide/KJ476722.1?report=genbank&log$=nucltop&blast_rank=2&RID=VUMP54MM013) | 99.86 | 100 |
| VKC_7 E1 | *Staphylococcus cohnii* | LT963440.1 | 96.77 | 100 |
| VKC_7 E2 | *Staphylococcus epidermidis* | [LN681574.1](https://www.ncbi.nlm.nih.gov/nucleotide/LN681574.1?report=genbank&log$=nucltop&blast_rank=1&RID=VUMYCG6K013) | 98.34 | 100 |
| VKC_ 8E1 | *Staphylococcus epidermidis* | [NR_113957.1](https://www.ncbi.nlm.nih.gov/nucleotide/NR_113957.1?report=genbank&log$=nucltop&blast_rank=50&RID=VUXWC0TT016) | 98.46 | 100 |
| VKC_ 8E2 | *Staphylococcus epidermidis* | CP035288.1 | 97.93 | 100 |
| VKC_9 E1 | *Staphylococcus haemolyticus* | [LC383923.1](https://www.ncbi.nlm.nih.gov/nucleotide/LC383923.1?report=genbank&log$=nucltop&blast_rank=1&RID=VUN5NTKD013) | 99.1 | 100 |
| VKC_9E2 | *Exiguobacterium aurantiacum* | [NR_043478.1](https://www.ncbi.nlm.nih.gov/nucleotide/NR_043478.1?report=genbank&log$=nucltop&blast_rank=1&RID=VUNEY89M013) | 99.8 | 100 |
| VKC_9E3 | *Staphylococcus epidermidis* | [CP035288.1](https://www.ncbi.nlm.nih.gov/nucleotide/CP035288.1?report=genbank&log$=nucltop&blast_rank=6&RID=VUNHEWCW013) | 99.18 | 100 |
| VKC_9E4 | *Staphylococcus epidermidis* | [CP035288.1](https://www.ncbi.nlm.nih.gov/nucleotide/CP035288.1?report=genbank&log$=nucltop&blast_rank=2&RID=VUNRFJ0F013) | 99.93 | 100 |
| VKC_10 E1 | *Kytococcus schroeteri* | [MT760114.1](https://www.ncbi.nlm.nih.gov/nucleotide/MT760114.1?report=genbank&log$=nucltop&blast_rank=2&RID=VUNUP9DV013) | 99.11 | 100 |
| VKC_10 E2 | *Staphylococcus haemolyticus* | [KT989857.1](https://www.ncbi.nlm.nih.gov/nucleotide/KT989857.1?report=genbank&log$=nucltop&blast_rank=19&RID=VUP1C4WF013) | 98.6 | 100 |
| VKC_11 E1 | *Staphylococcus haemolyticus* | LC383923.1 | 99.31 | 100 |
| VKC_11 E3 | *Staphylococcus haemolyticus* | [NR_113345.1](https://www.ncbi.nlm.nih.gov/nucleotide/NR_113345.1?report=genbank&log$=nucltop&blast_rank=3&RID=VUXWC0TT016) | 99.93 | 100 |
| VKC_12 E1 | *Staphylococcus epidermidis* | [CP035288.1](https://www.ncbi.nlm.nih.gov/nucleotide/CP035288.1?report=genbank&log$=nucltop&blast_rank=2&RID=VUPH6874013) | 99.61 | 100 |
| VKC_12 E2 | *Staphylococcus epidermidis* | [CP035288.1](https://www.ncbi.nlm.nih.gov/nucleotide/CP035288.1?report=genbank&log$=nucltop&blast_rank=2&RID=VUPPT0DD013) | 99.96 | 100 |
| VKC_13 E1 | *Staphylococcus haemolyticus* | [CP118979.1](https://www.ncbi.nlm.nih.gov/nucleotide/CP118979.1?report=genbank&log$=nucltop&blast_rank=11&RID=VUPU9MS7013) | 99.23 | 100 |
| VKC_14 E1 | *Micrococcus yunnanensis* | [NR_116578.1](https://www.ncbi.nlm.nih.gov/nucleotide/NR_116578.1?report=genbank&log$=nucltop&blast_rank=4&RID=VUR3B1S5013) | 97.02 | 100 |
| VKC_14 E2 | *Staphylococcus haemolyticus* | [KT989857.1](https://www.ncbi.nlm.nih.gov/nucleotide/KT989857.1?report=genbank&log$=nucltop&blast_rank=1&RID=VUR8HCAC013) | 99.2 | 100 |
| VKC_18 E1 | *Staphylococcus homnis* | [MF678883.1](https://www.ncbi.nlm.nih.gov/nucleotide/MF678883.1?report=genbank&log$=nucltop&blast_rank=2&RID=VUXMZ2XE013) | 99.77 | 100 |
| VKC_18 E2 | *Corynebacterium pilbarense* | [NR_116953.1](https://www.ncbi.nlm.nih.gov/nucleotide/NR_116953.1?report=genbank&log$=nucltop&blast_rank=1&RID=VUS6NURW013) | 97.1 | 100 |
| VKC_20 E1 | Achromobacter xylosoxidans | NR_113733.1 | 96.62 | 100 |
| VKC_20 E2 | *Achromobacter xylosoxidans* | [LN831029.1](https://www.ncbi.nlm.nih.gov/nucleotide/LN831029.1?report=genbank&log$=nucltop&blast_rank=2&RID=VUTNB69W016) | 98.79 | 100 |
| VKC_20 E7 | *Achromobacter xylosoxidans* | [NR_113733.1](https://www.ncbi.nlm.nih.gov/nucleotide/NR_113733.1?report=genbank&log$=nucltop&blast_rank=4&RID=VUTNB69W016) | 98.87 | 100 |
| VKC-21E1 | *Staphylococcus haemolyticus* | [LC383923.1](https://www.ncbi.nlm.nih.gov/nucleotide/LC383923.1?report=genbank&log$=nucltop&blast_rank=1&RID=VUTUJ6K6013) | 98.82 | 100 |
| VKC-21E2 | *Staphylococcus haemolyticus* | [CP118979.1](https://www.ncbi.nlm.nih.gov/nucleotide/CP118979.1?report=genbank&log$=nucltop&blast_rank=4&RID=VUXBW4E5016) | 99.93 | 100 |
| VKC_22 E1 | *Staphylococcus aureus* | [CP040998.1](https://www.ncbi.nlm.nih.gov/nucleotide/CP040998.1?report=genbank&log$=nucltop&blast_rank=2&RID=VUU3NEDK013) | 98.49 | 100 |
| VKC_22 E2 | *Staphylococcus aureus* | [CP040998.1](https://www.ncbi.nlm.nih.gov/nucleotide/CP040998.1?report=genbank&log$=nucltop&blast_rank=2&RID=VUTYE54W016) | 98.29 | 100 |
| VKC_23 E1 | *Staphylococcus epidermidis* | [AP019721.1](https://www.ncbi.nlm.nih.gov/nucleotide/AP019721.1?report=genbank&log$=nucltop&blast_rank=1&RID=VUU6YRTC016) | 98.48 | 100 |
| VKC_23 E2 | *Staphylococcus epidermidis* | [LN681574.1](https://www.ncbi.nlm.nih.gov/nucleotide/LN681574.1?report=genbank&log$=nucltop&blast_rank=1&RID=VUUBW1Z8016) | 94.85 | 100 |
| VKC_23 E3 | *Cutibacterium acnes* | [CP003084.1](https://www.ncbi.nlm.nih.gov/nucleotide/CP003084.1?report=genbank&log$=nucltop&blast_rank=2&RID=VUUWCSGA016) | 91.44 | 100 |
| VKC_24 E1 | *Staphylococcus epidermidis* | [LN681574.1](https://www.ncbi.nlm.nih.gov/nucleotide/LN681574.1?report=genbank&log$=nucltop&blast_rank=1&RID=VUUZU9WJ016) | 91.81 | 100 |
| VKC_2AT1 | *Staphylococcus aureus* | [CP040998.1](https://www.ncbi.nlm.nih.gov/nucleotide/CP040998.1?report=genbank&log$=nucltop&blast_rank=2&RID=VUV58G09016) | 99.87 | 100 |
| VKC_2AT2 | *Exiguobacterium aurantiacum* | [NR_043478.1](https://www.ncbi.nlm.nih.gov/nucleotide/NR_043478.1?report=genbank&log$=nucltop&blast_rank=1&RID=VUVEJTTP016) | 99 | 100 |
| VKC_2AT3 | *Corynebacterium aurimucosum* | [CP071874.1](https://www.ncbi.nlm.nih.gov/nucleotide/CP071874.1?report=genbank&log$=nucltop&blast_rank=1&RID=VUX3HET9016) | 99.81 | 100 |
| VKC_6AT1 | *Staphylococcus hominis* | [MF678884.1](https://www.ncbi.nlm.nih.gov/nucleotide/MF678884.1?report=genbank&log$=nucltop&blast_rank=2&RID=VUVKDST0013) | 100 | 100 |
| VKC_6AT2 | *Staphylococcus haemolyticus* | [CP118979.1](https://www.ncbi.nlm.nih.gov/nucleotide/CP118979.1?report=genbank&log$=nucltop&blast_rank=13&RID=VUVRVYT3013) | 98.74 | 100 |
| VKC_6AT3 | *Bacillus altitudinis* | [MZ276300.1](https://www.ncbi.nlm.nih.gov/nucleotide/MZ276300.1?report=genbank&log$=nucltop&blast_rank=4&RID=VUWA1X96016) | 98.97 | 100 |
| VKC_6AT4 | *Staphylococcus hominis* | [MF678883.1](https://www.ncbi.nlm.nih.gov/nucleotide/MF678883.1?report=genbank&log$=nucltop&blast_rank=2&RID=VUWJFPW4013) | 99.79 | 100 |
| VKC_9AT | *Staphylococcus hominis* | [NR_036956.1](https://www.ncbi.nlm.nih.gov/nucleotide/NR_036956.1?report=genbank&log$=nucltop&blast_rank=1&RID=VUWJFPW4013) | 99.89 | 100 |
| VKC_16AT1 | *Staphylococcus epidermidis* | [NR_113957.1](https://www.ncbi.nlm.nih.gov/nucleotide/NR_113957.1?report=genbank&log$=nucltop&blast_rank=2&RID=VUWSWCPE013) | 97.2 | 100 |
| VKC_16AT2 | *Staphylococcus epidermidis* | [CP035288.1](https://www.ncbi.nlm.nih.gov/nucleotide/CP035288.1?report=genbank&log$=nucltop&blast_rank=4&RID=VUWVB36D013) | 97.25 | 100 |
| VKC_16AT3 | *Staphylococcus epidermidis* | [KT989845.1](https://www.ncbi.nlm.nih.gov/nucleotide/KT989845.1?report=genbank&log$=nucltop&blast_rank=6&RID=VUX7NF80016) | 99.72 | 100 |

Table 2: BLAST analysis results of the HC isolates nearest neighbors in the database and their percentage relatedness.

| CODE | NEAREST_NEIGHBOUR | ACCESSION_NUMBER | SIMILARITY  (%) | QUERY_COVERAGE (%) |
| --- | --- | --- | --- | --- |
| HC_38t1 | *Cutibacterium acnes* | [CP023676.1](https://www.ncbi.nlm.nih.gov/nucleotide/CP023676.1?report=genbank&log$=nucltop&blast_rank=1&RID=VV4E50U0013) | 97.14 | 100 |
| HC_38t2 | *Cutibacterium acnes* | [CP023676.1](https://www.ncbi.nlm.nih.gov/nucleotide/CP023676.1?report=genbank&log$=nucltop&blast_rank=1&RID=VV64X1SK016) | 99.93 | 100 |
| HC_39t1 | *Cutibacterium acnes* | [CP023676.1](https://www.ncbi.nlm.nih.gov/nucleotide/CP023676.1?report=genbank&log$=nucltop&blast_rank=1&RID=VV4YK893016) | 99.1 | 100 |
| HC_39t2 | *Staphylococcus epidermidis* | [AP019721.1](https://www.ncbi.nlm.nih.gov/nucleotide/AP019721.1?report=genbank&log$=nucltop&blast_rank=5&RID=VV61GDWN013) | 99.93 | 100 |
| HC_39t3 | *Staphylococcus epidermidis* | [CP035288.1](https://www.ncbi.nlm.nih.gov/nucleotide/CP035288.1?report=genbank&log$=nucltop&blast_rank=2&RID=VV58XHYV016) | 99.51 | 100 |
| HC_39t4 | *Staphylococcus epidermidis* | [LN681574.1](https://www.ncbi.nlm.nih.gov/nucleotide/LN681574.1?report=genbank&log$=nucltop&blast_rank=1&RID=VV6J3D64013) | 100 | 100 |
| HC_41t1 | *Staphylococcus epidermidis* | [AP019721.1](https://www.ncbi.nlm.nih.gov/nucleotide/AP019721.1?report=genbank&log$=nucltop&blast_rank=5&RID=VV58XHYV016) | 99.44 | 100 |
| HC_41t2 | *Cutibacterium acnes* | [CP023676.1](https://www.ncbi.nlm.nih.gov/nucleotide/CP023676.1?report=genbank&log$=nucltop&blast_rank=1&RID=VV6C32ZB016) | 99.93 | 100 |
| HC_41t3 | *Staphylococcus epidermidis* | [AP019721.1](https://www.ncbi.nlm.nih.gov/nucleotide/AP019721.1?report=genbank&log$=nucltop&blast_rank=1&RID=VV5RKWU4016) | 99.49 | 100 |
| HC_41t4 | *Cutibacterium acnes* | [CP023676.1](https://www.ncbi.nlm.nih.gov/nucleotide/CP023676.1?report=genbank&log$=nucltop&blast_rank=1&RID=VV6PSB26016) | 99.93 | 100 |
| HC_41t5 | *Cutibacterium acnes* | [CP025934.1](https://www.ncbi.nlm.nih.gov/nucleotide/CP025934.1?report=genbank&log$=nucltop&blast_rank=2&RID=VV6WBGJ3016) | 100 | 100 |
| HC_42t1 | *Cutibacterium acnes* | CP023676.1 | 99.93 | 100 |
| HC_42t2 | *Micrococcus yunnanensis* | [NR_116578.1](https://www.ncbi.nlm.nih.gov/nucleotide/NR_116578.1?report=genbank&log$=nucltop&blast_rank=1&RID=VVAXDKXP016) | 100 | 100 |
| HC_42t3 | *Cutibacterium acnes* | [NR_040847.1](https://www.ncbi.nlm.nih.gov/nucleotide/NR_040847.1?report=genbank&log$=nucltop&blast_rank=3&RID=VVB0ZGVR016) | 99.93 | 100 |
| HC_43t1 | *Staphylococcus epidermidis* | [CP035288.1](https://www.ncbi.nlm.nih.gov/nucleotide/CP035288.1?report=genbank&log$=nucltop&blast_rank=8&RID=VV737MYZ013) | 97.4 | 100 |
| HC_43t2 | *Staphylococcus epidermidis* | [CP035288.1](https://www.ncbi.nlm.nih.gov/nucleotide/CP035288.1?report=genbank&log$=nucltop&blast_rank=4&RID=VV771FXT013) | 99.1 | 100 |
| HC_44t2 | *Staphylococcus epidermidis* | [CP035288.1](https://www.ncbi.nlm.nih.gov/nucleotide/CP035288.1?report=genbank&log$=nucltop&blast_rank=2&RID=VX5KA7DM013) | 100 | 100 |
| HC_44t3 | *Staphylococcus epidermidis* | [AP019721.1](https://www.ncbi.nlm.nih.gov/nucleotide/AP019721.1?report=genbank&log$=nucltop&blast_rank=1&RID=VVB3VRZ9013) | 98.59 | 100 |
| HC_45t1 | *Staphylococcus epidermidis* | [CP035288.1](https://www.ncbi.nlm.nih.gov/nucleotide/CP035288.1?report=genbank&log$=nucltop&blast_rank=2&RID=VVBM0ZNX013) | 100 | 100 |
| HC_46t1 | *Staphylococcus epidermidis* | [AP019721.1](https://www.ncbi.nlm.nih.gov/nucleotide/AP019721.1?report=genbank&log$=nucltop&blast_rank=5&RID=VVBPJTSY013) | 100 | 100 |
| HC_46t2 | *Staphylococcus epidermidis* | [KT989845.1](https://www.ncbi.nlm.nih.gov/nucleotide/KT989845.1?report=genbank&log$=nucltop&blast_rank=6&RID=VVBPJTSY013) | 99.72 | 100 |
| HC_46t4 | *Staphylococcus epidermidis* | [AP019721.1](https://www.ncbi.nlm.nih.gov/nucleotide/AP019721.1?report=genbank&log$=nucltop&blast_rank=1&RID=VV7J9NKX016) | 99.31 | 100 |
| HC_49t1 | *Staphylococcus epidermidis* | [CP035288.1](https://www.ncbi.nlm.nih.gov/nucleotide/CP035288.1?report=genbank&log$=nucltop&blast_rank=2&RID=VVBVZ5Y5013) | 96.98 | 100 |
| HC_49t2 | *Staphylococcus epidermidis* | [AP019721.1](https://www.ncbi.nlm.nih.gov/nucleotide/AP019721.1?report=genbank&log$=nucltop&blast_rank=2&RID=VVBYF9Z7016) | 99.2 | 100 |
| HC_50t1 | *Staphylococcus epidermidis* | [LN681574.1](https://www.ncbi.nlm.nih.gov/nucleotide/LN681574.1?report=genbank&log$=nucltop&blast_rank=1&RID=VVC14Z93013) | 98.41 | 100 |
| HC_50t2 | *Staphylococcus epidermidis* | [CP035288.1](https://www.ncbi.nlm.nih.gov/nucleotide/CP035288.1?report=genbank&log$=nucltop&blast_rank=2&RID=VV7RCCBK016) | 98.48 | 100 |
| HC_50t3 | *Staphylococcus epidermidis* | [CP035288.1](https://www.ncbi.nlm.nih.gov/nucleotide/CP035288.1?report=genbank&log$=nucltop&blast_rank=3&RID=VV7VPVB9013) | 99.44 | 100 |
| HC_51t3 | *Cutibacterium acnes* | [CP023676.1](https://www.ncbi.nlm.nih.gov/nucleotide/CP023676.1?report=genbank&log$=nucltop&blast_rank=1&RID=VV8107GH013) | 99.51 | 100 |
| HC_51t1 | *Cutibacterium acnes* | [NR_040847.1](https://www.ncbi.nlm.nih.gov/nucleotide/NR_040847.1?report=genbank&log$=nucltop&blast_rank=3&RID=VVC57D5S013) | 99.93 | 100 |
| HC_51t5 | *Cutibacterium acnes* | [CP023676.1](https://www.ncbi.nlm.nih.gov/nucleotide/CP023676.1?report=genbank&log$=nucltop&blast_rank=1&RID=VX5F9V4M013) | 99.93 | 100 |
| HC_53t1 | *Staphylococcus haemolyticus* | KT989857.1 | 97.31 | 100 |
| HC_53t2 | *Staphylococcus haemolyticus* | [CP035291.1](https://www.ncbi.nlm.nih.gov/nucleotide/CP035291.1?report=genbank&log$=nucltop&blast_rank=2&RID=VX33PRNB013) | 100 | 100 |
| HC_56t1 | *Staphylococcus haemolyticus* | [NR_113345.1](https://www.ncbi.nlm.nih.gov/nucleotide/NR_113345.1?report=genbank&log$=nucltop&blast_rank=3&RID=VX33PRNB013) | 99.93 | 100 |
| HC_56t2 | *Pseudomonas juntendi* | MK583463.1 | 100 | 100 |
| HC_57t1 | *Staphylococcus epidermidis* | CP035288.1 | 100 | 100 |
| HC_57t2 | *Micrococcus luteus strain* | [CP035298.1](https://www.ncbi.nlm.nih.gov/nucleotide/CP035298.1?report=genbank&log$=nucltop&blast_rank=9&RID=VX3XRUJX016) | 98.78 | 100 |
| HC_13t1 | *Staphylococcus epidermidis* | [NR_113957.1](https://www.ncbi.nlm.nih.gov/nucleotide/NR_113957.1?report=genbank&log$=nucltop&blast_rank=4&RID=VX3DJZX4013) | 99.93 | 100 |
| HC_59t2 | *Staphylococcus epidermidis* | [KT989845.1](https://www.ncbi.nlm.nih.gov/nucleotide/KT989845.1?report=genbank&log$=nucltop&blast_rank=6&RID=VX3DJZX4013) | 99.72 | 100 |
| HC_14t2 | *Staphylococcus aureus* | [CP040998.1](https://www.ncbi.nlm.nih.gov/nucleotide/CP040998.1?report=genbank&log$=nucltop&blast_rank=35&RID=VX42UJ0X013) | 96.99 | 100 |
| HC_60t1 | *Stutzerimonas stutzeri* | [MK583463.1](https://www.ncbi.nlm.nih.gov/nucleotide/MK583463.1?report=genbank&log$=nucltop&blast_rank=2&RID=VX4P594R013) | 98.46 | 100 |
| HC_60t2 | *Staphylococcus hominis* | [NR_036956.1](https://www.ncbi.nlm.nih.gov/nucleotide/NR_036956.1?report=genbank&log$=nucltop&blast_rank=25&RID=VX3RDUE1013) | 98.95 | 100 |
| HC_18t2 | *Staphylococcus epidermidis* | [CP035288.1](https://www.ncbi.nlm.nih.gov/nucleotide/CP035288.1?report=genbank&log$=nucltop&blast_rank=2&RID=VX48RRVA016) | 99.9 | 100 |

Table S2A: Percentage abundance of bacterial genera in different age groups

| **Age (years)** | **HC** | | **VKC** | |
| --- | --- | --- | --- | --- |
|  | **Genera** | **Abundance (%)** | **Genera** | **Abundance (%)** |
| 5-10 | *Staphylococcus* | 100 | *Achromobacter* | 25 |
|  |  |  | *Kytococcus* | 8.33 |
|  |  |  | *Micrococcus* | 8.33 |
|  |  |  | *Staphylococcus* | 58 |
| 11-15 | *Micrococcus* | 8.33 | *Bacillus* | 5.26 |
|  | *Pseudomonas* | 8.33 | *Cutibacterium* | 5.26 |
|  | *Staphylococcus* | 75 | *Exiguobacterium* | 5.26 |
|  | *Stutzerimonas* | 8.33 | *Kocuria* | 5.26 |
|  |  |  | *Micrococcus* | 5.26 |
|  |  |  | *Staphylococcus* | 73.68 |
| 16-20 | *Cutibacterium* | 42.3 | *Bacillus* | 5.54 |
|  | *Micrococcus* | 3.84 | *Corynebacterium* | 9.09 |
|  | *Staphylococcus* | 53.84 | *Exiguobacterium* | 5.54 |
|  |  |  | *Kocuria* | 5.54 |
|  |  |  | *Micrococcus* | 5.54 |
|  |  |  | *Staphylococcus* | 68.18 |
|  |  |  | *Stutzerimonas* | 5.54 |

Table S2B: Percentage abundance of bacterial species in different VKC types

| **Bacterial species** | **Limbal (%)** | **Palpebral (%)** | **Mixed (%)** |
| --- | --- | --- | --- |
| *Achromobacter xylosoxidans* | 13 | 0 | 0 |
| *Bacillus altitudinis* | 4.34 | 0 | 0 |
| *Bacillus licheniformis* | 4.34 | 0 | 0 |
| *Corynebacterium aurimucosum* | 0 | 0 | 5.55 |
| *Corynebacterium pilbarense* | 0 | 7.14 | 0 |
| *Cutibacterium acnes* | 0 | 7.14 | 0 |
| *Exiguobacterium aurantiacum* | 0 | 0 | 11.11 |
| *Kocuria rhizophila* | 4.34 | 0 | 5.55 |
| *Kytococcus schroeteri* | 4.34 | 0 | 0 |
| *Micrococcus yunnanensis* | 8.69 | 7.14 | 0 |
| *Staphylococcus aureus* | 8.69 | 0 | 16.66 |
| *Staphylococcus cohnii* | 0 | 7.14 | 0 |
| *Staphylococcus epidermidis* | 13 | 57.4 | 38.88 |
| *Staphylococcus haemolyticus* | 30.43 | 0 | 16.66 |
| *Staphylococcus homnis* | 8.69 | 7.14 | 5.55 |
| *Stutzerimonas stutzeri* | 0 | 7.14 | 0 |

Table S3A: Susceptibility of bacterial isolates to antibiotics in VKC group

| **Antimicrobial agent** | **Gentamicin (µg/ml)** | **Amikacin**  **(µg/ml)** | **Tobramycin**  **(µg/ml)** | **Streptomycin**  **(µg/ml)** | **Ciprofloxacin**  **(µg/ml)** | **Moxifloxacin**  **(µg/ml)** | **Ofloxacin**  **(µg/ml)** | **Ampicillin**  **(µg/ml)** | **Clindamycin**  **(µg/ml)** | **Cefuroxime**  **(µg/ml)** | **Ceftazidime**  **(µg/ml)** | **Cefazolin**  **(µg/ml)** | **Triamcinolone(µg/ml)** | **Azithromycin**  **(µg/ml)** | **Vancomycin**  **(µg/ml)** |
| --- | --- | --- | --- | --- | --- | --- | --- | --- | --- | --- | --- | --- | --- | --- | --- |
| *S.haemolyticus* (VKC-21 E1) | 64 (R) | 8(S) | >64(R) | 2 | 4 (R) | 0.5(S) | 4 (R) | 0.5(R) | >64(R) | 8 (S) | 32(R) | 2(S) | >64 | 1(S) | 2(S) |
| *S.cohnii* (VKC-7E1) | 32 (R) | 4(S) | 64(R) | 4 | 8(R) | 0.5(S) | 16(R) | 16(R) | 32(R) | >64(R) | >64(R) | 32(R) | >64 | >64(R) | 4(S) |
| *S.haemolyticus* (VKC-9E1) | 64 (R) | 8(S) | >64(R) | 4 | 32(R) | 4(R) | 32(R) | 2(R) | 32(R) | 8(S) | 32(R) | 2(S) | >64 | >64(R) | 2(S) |
| *S. hominis* (VKC-6AT1) | 1(S) | 2(S) | 1(S) | 2 | 0.25(S) | 0.125(S) | 0.5(S) | 0.125(S) | 16(R) | 0.5(S) | 2 (S) | 0.5(S) | >64 | 0.5(S) | 0.5(S) |
| *S. homnis* (VKC-6AT4) | >64(R) | 64(R) | >64(R) | 2 | 1(S) | 0.125(S) | 1(S) | 4(R) | >64(R) | 32(R) | 64(R) | 4(S) | >64 | >64(R) | 4(S) |
| *S. epidermidis* (VKC-8E1) | 16(R) | 4(S) | 1(S) | 2 | 0.5(S) | 0.125(S) | 0.5(S) | 0.125(S) | 8(R) | 2(S) | 2(S) | 0.125(S) | >64 | 0.125 | 1(S) |
| *S.hominis* (VKC-9AT) | 0.25(S) | 4(S) | 2(S) | 2 | 0.125(S) | 0.125(S) | 0.5(S) | 0.5(R) | 4(R) | 8(S) | 8 (S) | 0.25(S) | >64 | 1(S) | 1(S) |
| *S.epidermidis* (VKC-16AT1) | 64(R) | 16 | >64(R) | 4 | 0.125(S) | 0.125(S) | 0.5(S) | 2(R) | 16(R) | 4(S) | 8 (S) | 1(S) | >64 | >64(R) | 4(S) |
| *S.epidermidis*(VKC-12E1) | >64(R) | 64 | >64(R) | 4 | 1(S) | 4(R) | 4(R) | 2(R) | >64(R) | 32(R) | 64(R) | 2(S) | >64 | 64(R) | 16 |
| *S.haemolyticus*(VKC-6AT2) | 0.25(S) | 2(S) | 0.5(S) | 4 | 0.125(S) | 0.125(S) | 0.125(S) | 1(R) | >64(R) | 32(R) | 4 (S) | 4(S) | >64 | 1(S) | 1(S) |
| *S.haemolyticus*(VKC-14E2) | 32(R) | 4(S) | >64(R) | 2 | 4(R) | 0.125(S) | 2(S) | 0.25(S) | >64(R) | 4(S) | 16 (S) | 2(S) | >64 | 1(S) | 4(S) |
| *S.aureus*  (VKC-22 E2) | 16(R) | 8(S) | 2(S) | 8 | 64(R) | 4(R) | 32(R) | 0.5(R) | 8(R) | 0.25 | 4 (S) | 0.125(S) | >64 | >64(R) | 2(S) |
| *S.aureus*  (VKC-2E3) | 1(S) | 4(S) | 4(R) | 16 | 32(R) | 2(R) | 16(R) | 8(R) | 4(R) | 64(R) | >64(R) | 32(R) | >64 | >64(R) | 4(S) |
| *S.aureus* (VKC-22E1) | 32(R) | >64 | 32(R) | 16 | 64(R) | 8(R) | 32(R) | 0.125(S) | 16(R) | 4(S) | 4 (S) | 0.25(S) | >64 | 16(R) | 4(S) |
| *S. aureus* (VKC-2AT1) | 4(R) | 8(S) | 8(R) | 4 | 2(R) | 8(R) | 2(S) | >64(R) | >64(R) | >64(R) | 0.5 (S) | >64(R) | >64 | 2(S) | >64(R) |
| *Exiguobacterium aurantiacum* (VKC-9E2) | 0.5 | 2 | 1 | 2 | 0.25 | 0.125 | 1 | 0.125 | 8 | 2 | 1 | 0.125 | >64 | 16 | 0.5 |
| *Bacillus altitudinis* (VKC-6AT3) | >64 | 32 | >64 | 16 | 0.5 (R) | 0.125 | 1 | 2 | >64(R) | 16 | 32 | 4 | >64 | >64 | 8(R) |
| *Bacillus licheniformis*(VKC-AE1) | 0.25 | 1 | 0.5 | 4 | 0.125(S) | 0.125 | 0.25 | 0.5 | 16(R) | 16 | 32 | 2 | >64 | 0.5 | 2(R) |
| *Achromobacter xylosoxidans*(VKC-20E1) | 0.5 | 2 | 0.5 | 2 | 0.25 | 0.125 | 1 | 0.125 | 8 | 1 | 8 | 0.5 | >64 | >64 | 8 |
| *Achromobacter xylosoxidans*(VKC-20E7) | 2 | 2 | 2 | 16 | 4 | 4 | 2 | >64 | >64 | >64 | 8 | >64 | >64 | 0.5 | >64 |
| *Corynebacterium*(AC-2AT3) | >64 | 16 | >64 | 8 | 1(R) | 0.125(S) | 2 | 1 | 8(R) | 8 | 32 | 2 | >64 | 64 | 4(R) |
| *Corynebacterium*(VKC-18E2) | 2 | 2 | 1 | 4 | 2(R) | 4(R) | 8 | 64 | >64(R) | >64 | 2 | >64 | >64 | 2 | >64(R) |
| *Micrococcus yunnanensis*(VKC-5E2) | 2 | 16 | 4 | 4 | 8 | 1 | 16 | 0.25 | 16 | 0.5 | 4 | 0.25 | >64 | >64 | 4 |
| *Micrococcus yunnanensis*(VKC-14E1) | 2 | 0.125 | 0.5 | 0.5 | 32 | 0.125 | 16 | 0.125 | >4 | 2 | 4 | 0.25 | >64 | >64 | 2 |
| *Stutzerimonas stutzeri*(VKC-5E3) | 0.5 | 2 | 1 | 4 | 0.125(S) | 0.125 | 0.125 | 1 | >64 | >64 | 0.5 | 32 | >64 | 0.25 | >64 |
| *Cutibacterium acnes* (VKC-23E3) | 4 | 2 | 2 | 1 | 8 | 1 | 0.125 | 0.125(S) | >64(R) | O.125 | 8 | 0.25 | >64 | >64 | 4(R) |
| *Kytococcus schroeteri* (VKC-10E1) | 32 | 8 | 64 | 4 | 4 | 2 | 8 | 1 | 16 | 4 | 16 | 1 | >64 | >64 | 4 |

Table S3B: Susceptibility of bacterial isolates to antibiotics in HC group

| Antimicrobial agent | Gentamicin  (µg/ml) | Amikacin(µg/ml) | Tobramycin  (µg/ml) | Streptomycin  (µg/ml) | Ciprofloxacin  (µg/ml) | Moxifloxacin  (µg/ml) | Ofloxacin  (µg/ml) | Ampicillin  (µg/ml) | Clindamycin  (µg/ml) | Cefuroxime  (µg/ml) | Ceftazidime  (µg/ml) | Cefazolin  (µg/ml) | Triamcinolone  (µg/ml) | Azithromycin  (µg/ml) | Vancomycin  (µg/ml) |
| --- | --- | --- | --- | --- | --- | --- | --- | --- | --- | --- | --- | --- | --- | --- | --- |
| *S.epidermidis* (HC-49t2) | 0.125(S) | 4(S) | 1(S) | 0.125 | 0.125(S) | 0.125(S) | 0.125(S) | 0.125(S) | 0.125(S) | 0.125(S) | 0.5(S) | 0.125(S) | >64 | 0.125(S) | 0.125(S) |
| *S.epidermidis* (HC-46t1) | 2(S) | 8(S) | 2(S) | 8 | 0.25(S) | 0.125(S) | 0.25(S) | 0.25(S) | 2(S) | 1(S) | 16(S) | 0.25(S) | >64 | 2(S) | 1(S) |
| *S.haemolyticus* (HC-53t1) | 8(I) | 4(S) | 16(R) | 64 | 0.5(S) | 0.25(S) | 2(S) | 16 (R) | 64(R) | >64(R) | >64(R) | >64(R) | >64 | 64(R) | 4(S) |
| *S.haemolyticus.*(HC-53t2) | 8(I) | 2(S) | 16 (R) | 64 | 0.5(S) | 0.125(S) | 0.5(S) | 16 (R) | 64(R) | >64(R) | >64(R) | >64(R) | >64 | 64(R) | 4(S) |
| *S.epidermidis* (HC-46t2) | 4 (S) | 16(S) | 0.125(S) | 4 | 0.5(S) | 0.125(S) | 1(S) | 8(R) | 32(R) | 16(S) | 64(R) | 4(S) | >64 | 64(R) | 8(S) |
| *S.epidermidis* (HC-18t2) | 32 (R) | 2(S) | 2(S) | 2 | 0.5(S) | 0.125(S) | 0.5(S) | 0.25(S) | 2(S) | 0.125(S) | 4(S) | 0.25(S) | >64 | >64(R) | 0.5(S) |
| *S.epidermidis* (HC-13t1) | >64(R) | >64(R) | >64(R) | 16 | 0.25(S) | 0.125(S) | 0.5(S) | 8(R) | 1(S) | 16(S) | 64(R) | 4(S) | >64 | 64(R) | 4(S) |
| *S.epidermidis* (HC-59t2) | 4(S) | 4(S) | 8(S) | 32 | 0.25(S) | 0.125(S) | 2(S) | 16(R) | 64(R) | >64(R) | 64(R) | 64(R) | >64 | 1(S) | 2(S) |
| *S.epidermidis* (HC-50t3) | 2(S) | 4(S) | 2(S) | 2 | 0.25(S) | 0.125(S) | 0.5(S) | 0.125(S) | 1(S) | 0.5(S) | 4(S) | 0.125(S) | >64 | 64(R) | 4(S) |
| *Cutibacterium acnes*(HC-42t3) | 8 | 1 | 0.5 | 0.5 | 0.5 | 0.125 | 0.5 | 0.125(S) | 64(R) | 1 | 16 | 1 | >64 | 1 | 4(R) |
| *Cutibacterium acnes*(HC-51t1) | >64 | 8 | 1 | 4 | 0.5 | 0.125 | 1 | 2 | 32(R) | 16 | 32 | 32 | >64 | 64 | 2(R) |
| *Cutibacterium acnes*(HC-51t5) | 0.5 | 4 | 1 | 1 | 0.125 | 0.125 | 0.125 | 0.125(S_ | 64(R) | 8 | 32 | 0.25 | >64 | 0.5 | 0.5(S) |
| *Cutibacterium acnes (*HC-38t1) | 0.125 | 2 | 2 | 1 | 0.125 | 0.125 | 0.125 | 0.125(S) | 0.125(S) | 0.25 | 1 | 0.5 | >64 | 0.125 | 0.5(S) |
| *Cutibacterium acnes*(HC-39t1) | 1 | 4 | 2 | 2 | 0.25 | 0.125 | 0.5 | 0.125(S) | 16(R) | 0.5 | 8 | 0.5 | >64 | 0.5 | 4(R) |
| *Micrococcus yunnanensis* (HC-42t2) | 1 | 2 | 1 | 2 | 0.125 | 0.125 | 0.125 | 0.125 | 64 | 8 | 32 | 0.25 | >64 | 0.5 | 1 |

Table S4A: Differences in Bacterial Genera between Males and Females in VKC

| Genera | Abundance in Males (%) | Abundance in Females (%) | p-value |
| --- | --- | --- | --- |
| [Staphylococcus](https://www.ncbi.nlm.nih.gov/Taxonomy/Browser/wwwtax.cgi?mode=Undef&id=1279&lvl=3&lin=f&keep=1&srchmode=1&unlock) | 64.10 | 78.57 | 0.001 |
| [Micrococcus](https://www.ncbi.nlm.nih.gov/Taxonomy/Browser/wwwtax.cgi?mode=Undef&id=1269&lvl=3&keep=1&srchmode=1&unlock) | 7.69 | 0 | 0.001 |
| [Achromobacter](https://www.ncbi.nlm.nih.gov/Taxonomy/Browser/wwwtax.cgi?mode=Undef&id=222&lvl=3&lin=f&keep=1&srchmode=1&unlock) | 7.69 | 0 | 0.001 |
| [Bacillus](https://www.ncbi.nlm.nih.gov/Taxonomy/Browser/wwwtax.cgi?mode=Undef&id=1386&lvl=3&lin=f&keep=1&srchmode=1&unlock) | 5.12 | 0 | 0.002 |
| [Kocuria](https://www.ncbi.nlm.nih.gov/Taxonomy/Browser/wwwtax.cgi?mode=Undef&id=57493&lvl=3&lin=f&keep=1&srchmode=1&unlock) | 2.56 | 7.14 | 0.005 |
| [Exiguobacterium](https://www.ncbi.nlm.nih.gov/Taxonomy/Browser/wwwtax.cgi?mode=Undef&id=33986&lvl=3&lin=f&keep=1&srchmode=1&unlock) | 2.56 | 7.14 | 0.005 |
| [Corynebacterium](https://www.ncbi.nlm.nih.gov/Taxonomy/Browser/wwwtax.cgi?mode=Undef&id=1716&lvl=3&lin=f&keep=1&srchmode=1&unlock) | 2.56 | 7.14 | 0.005 |
| [Stutzerimonas](https://www.ncbi.nlm.nih.gov/Taxonomy/Browser/wwwtax.cgi?mode=Undef&id=2901164&lvl=3&lin=f&keep=1&srchmode=1&unlock) | 2.56 | 0 | 0.106 |
| [Cutibacterium](https://www.ncbi.nlm.nih.gov/Taxonomy/Browser/wwwtax.cgi?mode=Undef&id=1912216&lvl=3&lin=f&keep=1&srchmode=1&unlock) | 2.56 | 0 | 0.106 |
| [Kytococcus](https://www.ncbi.nlm.nih.gov/Taxonomy/Browser/wwwtax.cgi?mode=Undef&id=57499&lvl=3&lin=f&keep=1&srchmode=1&unlock) | 2.56 | 0 | 0.106 |

Table S4B: Differences in Bacterial Genera between Males and Females in HC

| Genus | Abundance in Males | Abundance in Females | p-value |
| --- | --- | --- | --- |
| [Staphylococcus](https://www.ncbi.nlm.nih.gov/Taxonomy/Browser/wwwtax.cgi?mode=Undef&id=1279&lvl=3&lin=f&keep=1&srchmode=1&unlock) | 60 | 75 | 0.001 |
| Cutibacterium | 26.66 | 25 | 0.265 |
| [Pseudomonas](https://www.ncbi.nlm.nih.gov/Taxonomy/Browser/wwwtax.cgi?mode=Undef&id=286&lvl=3&keep=1&srchmode=1&unlock) | 3.33 | 0 | 0.029 |
| [Stutzerimonas](https://www.ncbi.nlm.nih.gov/Taxonomy/Browser/wwwtax.cgi?mode=Undef&id=2901164&lvl=3&lin=f&keep=1&srchmode=1&unlock) | 3.33 | 0 | 0.029 |
| [Micrococcus](https://www.ncbi.nlm.nih.gov/Taxonomy/Browser/wwwtax.cgi?mode=Undef&id=1269&lvl=3&keep=1&srchmode=1&unlock) | 6.66 | 0 | 0.001 |

Table S4C: Differences in biofilm formation ability between Males and Females in healthy and VKC

| Sl.No | Healthy | | | VKC | | |
| --- | --- | --- | --- | --- | --- | --- |
|  | Male % | Female % | p-value | Male % | Female % | p-value |
| Non-biofilm | 37.9 | 30.8 | 0.001 | 25.6 | 14.3 | 0.001 |
| Weak | 41.4 | 53.8 | 0.001 | 25.6 | 57.1 | 0.001 |
| moderate | 20.7 | 15.4 | 0.001 | 28.2 | 21.4 | 0.001 |
| High | 0 | 0 | - |  |  |  |
